# Fermentation pH Modulates the Size Distributions and Functional Properties of *Gluconobacter albidus* TMW 2.1191 Levan

**DOI:** 10.3389/fmicb.2017.00807

**Published:** 2017-05-04

**Authors:** Tharalinee Ua-Arak, Frank Jakob, Rudi F. Vogel

**Affiliations:** Lehrstuhl für Technische Mikrobiologie, Technische Universität MünchenFreising, Germany

**Keywords:** *Gluconobacter*, exopolysaccharide, levan, field-flow fractionation, light scattering, optimization, gluten-free

## Abstract

Bacterial levan has gained an increasing interest over the last decades due to its unique characteristics and multiple possible applications. Levan and other exopolysaccharides (EPSs) production are usually optimized to obtain the highest concentration or yield while a possible change of the molecular size and mass during the production process is mostly neglected. In this study, the molar mass and radius of levan samples were monitored during fermentations with the food-grade, levan-producing acetic acid bacterium *Gluconobacter (G.) albidus* TMW 2.1191 in shake flasks (without pH control) and bioreactors (with pH control at 4.5, 5.5 and 6.5, respectively). In uncontrolled fermentations, the levan size/molar mass continuously decreased concomitantly with the continuous acidification of the nutrient medium. On the contrary, the amount, molar mass and size of levan could be directly influenced by controlling the pH during fermentation. Using equal initial substrate amounts, the largest weight average molar mass and geometric radius of levan were observed at constant pH 6.5, while the highest levan concentration was obtained at constant pH 4.5. Since there is a special demand to find suitable hydrocolloids from food-grade bacteria to develop novel gluten-free (GF) products, these differently produced levans were used for baking of GF breads, and the best quality improvement was obtained by addition of levan with the highest mass and radius. This work, therefore, demonstrates for the first time that one bacterial strain can produce specific high molecular weight fractions of one EPS type, which differ in properties and sizes among each other in dependence of the controllable production conditions.

## Introduction

Levan consists of fructose monomers linked by β-(2,6) glycosidic bonds with possible β-(2,1) branches. It is a homopolysaccharide (fructan) that can be synthesized by some plant species usually with lower degree of polymerization (DP < 100) or extracellularly by several microorganisms generally with higher molecular masses (Öner et al., 2016). Due to its unique characteristics such as low intrinsic viscosity (Arvidson et al., 2006) and high water solubility (Han and Clarke, 1990), levan can be used in several applications ranging from food and feed (prebiotics, stabilizer, fat substitute), cosmetics (whitener, moisturizer), to pharmaceuticals (anti-oxidant, anti-inflammatory, anti-cancer activities) industries (Moscovici, 2015; Srikanth et al., 2015; Öner et al., 2016). For bacterial levan synthesis, levansucrases (E.C. 2.4.1.10, sucrose: 2-6-β-D-fructan 6-β-D-fructosyltransferase) are the enzymes responsible for the catalysis of polyfructose chains from sucrose substrate. In addition to their fructosyltransferase activity, which results in the formation of levan and/or oligosaccharides (having fructan chain or sucrose as acceptors, respectively), levansucrases also exhibit a sucrose hydrolysis activity when water is used as an acceptor (Yanase et al., 1991).

Several bacterial strains are reported to produce levan from sucrose, including gram-positive bacteria such as *Bacillus (B.) subtilis* (Tanaka et al., 1978), *Paenibacillus polymyxa* (Liang and Wang, 2015), *Leuconostoc citreum* (Han et al., 2016), and *Streptococcus* sp. (Simms et al., 1990), and gram-negative bacteria such as *Gluconobacter (G.)* species (Jakob et al., 2012a), *Gluconacetobacter (Ga.) diazotrophicus* (Molinari and Boiardi, 2013), *Halomonas* sp. (Kucukasik et al., 2011), *Zymomonas (Z.) mobilis* (Yanase et al., 1991), and *Pseudomonas fluorescens* (Raza et al., 2012). In addition to the differences in final levan concentrations, bacterial levans also vary in their molecular sizes and masses, depending on the strains used. For example, *B. subtilis* NATTO produced levan with two fractions of molecular weight (M_w_) less than 50 kDa and 0.5–2 MDa (Dos Santos et al., 2013; Wu et al., 2013), while levans isolated from different acetic acid bacteria (AAB) differed in molecular weights in dependence of the producer strains (Jakob et al., 2013). These variations of levan size from different producing strains were influential in the characteristics and function of the corresponding levan, as demonstrated in earlier studies for example, by the differences in the viscosity, rheological and food technological properties of levans produced by *Z. mobilis, Erwinia herbicola, B. subtilis* (Benigar et al., 2014) or AAB (Brandt et al., 2016), or by the changes in the antitumor activities (Calazans et al., 2000) and antiviral activities (Esawy et al., 2011) of levan with different molecular weights from *Z. mobilis* and *Bacillus* sp., respectively.

Some environmental factors such as sucrose concentration, temperature, and agitation speed were reported to influence the ratios of short chain oligosaccharides and high molecular weight levans (Euzenat et al., 1997; Abdel-Fattah et al., 2005; Wu et al., 2013; Santos-Moriano et al., 2015). In addition to the sucrose concentration, which was reported to control the M_w_ of levan synthesized by *B. subtilis* (Euzenat et al., 1997; Abdel-Fattah et al., 2005; Wu et al., 2013), Runyon et al. (2014) reported changes in M_w_ of high molecular weight levan isolated from *Z. mobilis* in different pH environments (Runyon et al., 2014). Since the pH during the fermentation of bacteria such as AAB and lactic acid bacteria usually reduces due to acid formations, the molar mass of levan produced by these strains could also be influenced by the pH changes during the fermentation process. In our previous works we have found that the molecular size and mass of levan produced *in situ* by *Gluconobacter albidus* reduced during the fermentation process (Ua-Arak et al., 2016, 2017), accordingly, it was suspected that this change might be due to the pH reduction during the AAB fermentation. Since some AAB strains are able to produce extraordinary high molecular weight levans (Jakob et al., 2013), a better understanding on the changes of these levans during fermentations can provide more information on the targeted production of levan from AAB for suitable applications that have not been explored before.

The aim of this study was therefore to elucidate the changes of high M_w_ levan molecular size and mass during the levan production in batch culture by food-grade *G. albidus* TMW 2.1191 (isolated from kefir), which could be established for the customization of bacterial levan production or as starter culture in foods, and ultimately to obtain specific levans of desired size for various applications. To test and demonstrate the usefulness and functional differences of distinctively produced levans, we used these levans in equal amounts for baking of gluten-free (GF) breads as a model system to establish new possibilities for the development of novel foods for patients suffering from celiac disease (Ua-Arak et al., 2017).

## Materials and Methods

### Bacterial Strain and Culture Conditions

*Gluconobacter albidus* TMW 2.1191 isolated from water kefir (Gulitz et al., 2011) was grown aerobically at 30°C in sodium gluconate (NaG) medium (pH 6.2) consisted of (per liter) sodium gluconate (20 g), yeast extract (3 g), peptone from casein (2 g), glycerol (3 g), MgSO_4_.7H_2_O (0.2 g), mannitol (10 g), and agar (20 g) for agar plates (Adachi et al., 1979).

### Levan Production

Levan production in shake flask culture was performed at 30°C, 200 rpm for up to 48 h in 100-mL Erlenmeyer flasks containing 10 mL of NaG medium supplemented with 40 g/L sucrose, 7 g/L glucose and 10 g/L fructose with initial cell count of ca. 1 × 10^7^ CFU/mL. For pH control, batch fermentation was performed in a 1 L bioreactor (Biostat^®^ A, Sartorius Stedim Biotech GmbH, Germany) at 800 mL working volume using NaG medium containing 80 g/L sucrose for higher level of levan production. Pre-cultures (50 mL) were prepared in shake flasks (500 mL) to mid-exponential growth phase, centrifuged at 13000 *g* for 15 min and re-suspended in fresh NaG medium (5 mL) for inoculation at 0.5%. The fermentation was operated at 30°C with 600 rpm agitation and 1 vvm aeration for 48 h, using 3 M NaOH and 10% Antifoam B emulsion (A5757, Sigma-Aldrich, Germany) for pH and foaming controls, respectively.

### EPS Isolation

During fermentation, samples were drawn for the optical density (OD) at 600 nm, pH and levan measurements. The isolation and quantification of levan were performed following the common method of exopolysaccharide (EPS) purification which included cell removal, ethanol precipitation, dialysis, and lyophilization (Korakli et al., 2001; Notararigo et al., 2013; Torino et al., 2015). Cell culture was first centrifuged (13000 *g*, 15 min, 4°C) and the supernatant was incubated overnight at 4°C with two volumes of absolute ethanol (-20°C) before centrifugation using the same conditions to collect the precipitates. After air-drying, precipitates were re-dissolved in demineralized water (dH_2_O), dialyzed against dH_2_O for 48 h (MEMBRA-CEL, Serva Electrophoresis GmbH, Germany) and freeze dried for at least 24 h (FreezeZone 2.5 Plus, Labconco, USA) before weighing. The fructan (levan) type of the isolated substances was confirmed by fructose determination via HPLC analysis after acidic hydrolysis of the isolated samples as described previously (Ua-Arak et al., 2016, 2017).

### Structural Analysis

Analysis of EPS structure was performed by asymmetric flow field-flow fractionation (AF4) (Wyatt Technology, Germany) coupled to multi-angle laser light scattering (MALS) (Dawn Heleos II, Wyatt Technology, Germany). AF4-MALS was additionally coupled to UV (concentration) detection (Dionex Ultimate 3000, Thermo Fisher Scientific, USA) to calculate molar masses as described in Ua-Arak et al. (2016). The 10 kDa regenerated cellulose membranes (Superon GmbH, Germany) were used for separation with 50 mM NaNO_3_ (aq.) as eluent solution. Levan isolated from Section “EPS Isolation” was dissolved in dH_2_O to 0.1–0.33 g/L before being injected (100 μL) into the separation channel. At least two measurements were performed from each sample and the data were analyzed regarding geometric radii (MALS signals) and molar masses (MALS and UV signals). Preliminary optimization experiments for levan separation and characterization revealed levans to generate accurate UV signals at 400 nm. For calculation of the molar masses using UV concentration signals, the specific extinction coefficients of all isolated levans had therefore to be first measured and calculated at 400 nm. For this purpose, a concentration series (0.1–10 mg/mL levan; aqu.) of the respective isolated levans was prepared, from which the UV extinctions at 400 nm were measured using a FLOUstar Omega microplate reader (BMG Labtech, Ortenberg, Germany), respectively. The obtained values were used to finally calculate the specific extinction coefficients [mL/(mg⋅cm)] of the respective isolated levan samples [from the plot/slope of extinction_400__nm_ against specific concentration (mg/mL)], which again could be used to calculate the molar mass distributions using the sphere model for globular levans integrated in the ASTRA 6.1 software (Wyatt Technology, Germany) and a refractive index increment (dn/dc) value of 0.146 mL/g (in 50 mM NaNO_3_) for spherical levan (Jakob et al., 2013).

### Bread Baking and Analysis

Buckwheat breads (DY 200) were made from organic buckwheat flour (100 g) (Bauck GmbH & Co. KG, Rosche, Germany), tap water (100 g), salt (2 g), and instant dried yeast (3 g) (Fermipan^®^ Red, UK). For bread containing isolated levan [1% flour base addition, which was equivalent to 0.49% (w/w) of total dough weight], 1 g of freeze dried levan was first mixed with the dry ingredients before adding 99 g of water. Bread doughs were mixed at speed no.1 (10 s) and no. 5 (110 s) by a hand mixer (450W Bosch, Germany) before distributing into mini aluminum trays (50 g each). Afterward, they were rested for 45 min in a proofing chamber (30°C, 80% humidity) and baked for 15 min at 230°C in an oven (Wachtel Piccolo, Germany). Three bread loaves of each sample were made from one individual baking and three separated bakings were performed for each type of breads. Bread loaves were cooled for 2 h before analysis.

The analysis of bread volume and crumb hardness were modified from Konitzer et al. (2013). The specific volume [mL/g] of a bread loaf was measured in triplicate by a laser-based scanner (Volscan Profiler 300, Stable Micro Systems, UK). For the crumb hardness determination, the heels of the loaf of bread (10 mm thick) were first removed and then sliced into 15 mm thickness. Texture profile analysis (TPA) of the bread crumbs were performed by a texture analyzer (TA.XT.plus, Stable Micro Systems, UK) using a 20 mm diameter cylinder probe with a test speed of 0.50 mm/s. The force that the probe required to penetrate the bread slice to 7.0 mm was recorded and displayed as crumb hardness [N]. Four slices/bread of a total three breads per sample were analyzed in one individual baking.

### Statistical Analysis

Data were evaluated by one-way ANOVA using SigmaPlot (version 12.5, Systat Software Inc., USA). The Tukey’s honestly significant difference (HSD) was used to describe means at 5% significance level (*p* < 0.05).

## Results

### Levan Production in Shake Flasks without pH Control

**FIGURE 1:**
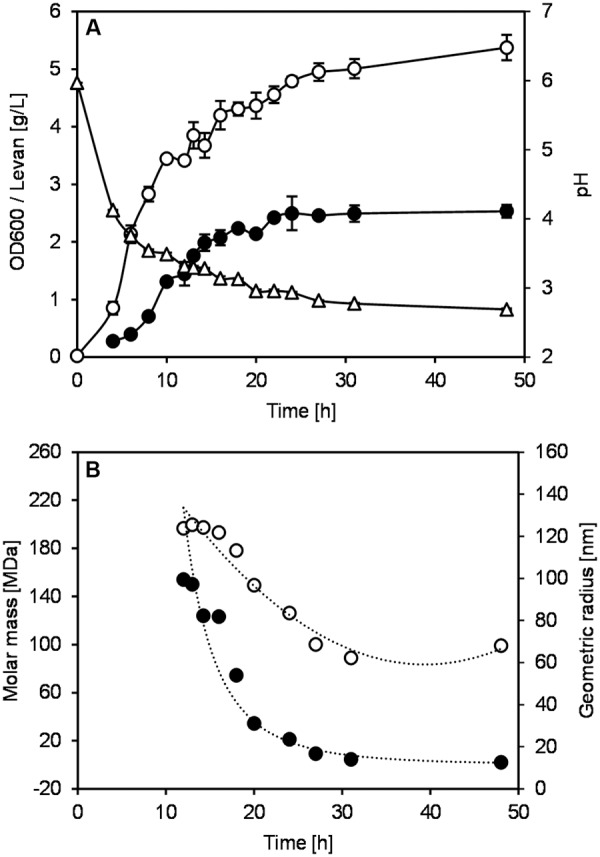
**Growth and levan production of *Gluconobacter albidus* in shake flasks without pH control. (A)** (∘) OD600, (Δ) pH, (●) levan. Change of levan structure during fermentation. **(B)** (●) molar mass, (∘) geometric radius. Data are average values ± SD (*n* = 3).

*Gluconobacter albidus* was cultivated in shake flasks for 48 h to observe the growth, pH and levan production without pH control (**Figure 1A**). A steady increase of OD was observed together with a constant reduction of pH from around 6 to 3 in the first 24 h. Levan production also increased in the same manner as OD, reaching stable values of ca. 2.5 g/L at around 22 h. Levan isolated at 12–48 h were analyzed by AF4 to observe the change of levan molecular sizes and masses during the fermentation process (**Figure 1B**). Although there was still an increase in the levan quantity from 12 to 22 h, the M_w_ and radius of levan particles decreased gradually until around 27 h before reaching constant size distributions. Comparing the levan particles from 12 to 31 h (pH 3.41 and 2.9, respectively), the weight average geometric radius (R_wgeo_) reduced from around 124 to 62 nm, while the weight average molar mass (M_w_) decreased from around 154 to 5 MDa. The polydispersity index (PDI) of levan particles was also decreasing toward monodispersity (Mw/Mn = 1), in which the Mw/Mn reduced from ca. 1.209 at 12 h to 1.005 at 31 h.

**FIGURE 2:**
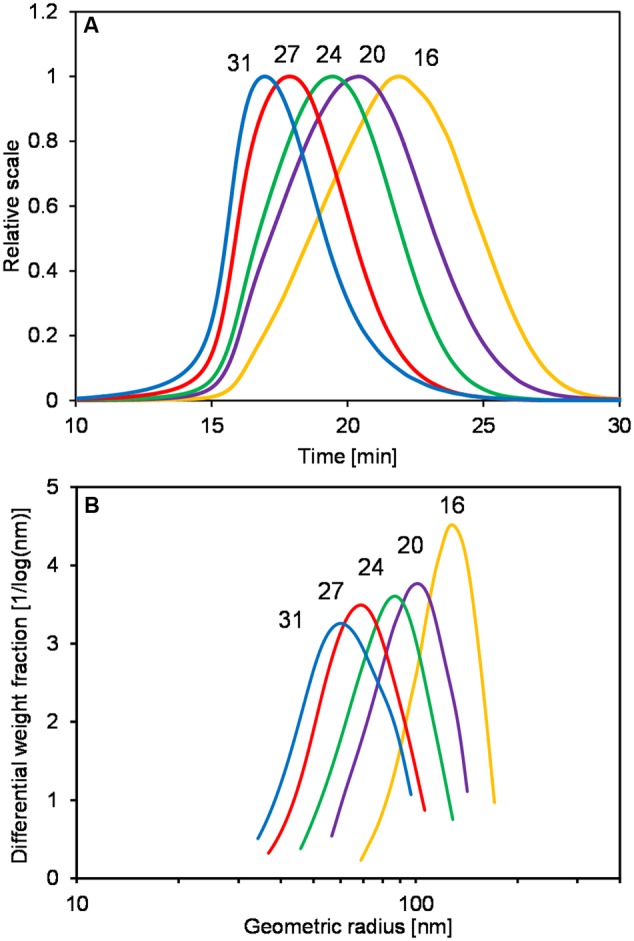
**Light scattering (LS) signals at 90° (A)** and size distributions of the geometric radius **(B)** of levan samples, which were produced without pH control and were isolated at different time points.

In addition to the determination of M_w_ and R_wgeo_ of levan samples, the reduction of levan size could be easily noticed first-hand by comparing the retention time of the light scattering (LS) signals of each sample. **Figure 2A** displays the LS signals at 90° of the selected levan samples from 16 to 31 h, where a shift of retention time could be observed. The retention time of the LS profiles changed from around 22 min in the levan isolated at 16 h to 17 min in the levan isolated at 31 h, indicating the smaller size of levan samples at later fermentation time. The molecular size distribution of levan particles from different time points (**Figure 2B**) further illustrated how the size of levan changed along the fermentation process. While size distributions of levan particles isolated at different time points were overlapping to a certain extent among each other, the main part of levan particles present in the individual samples varied, as displayed by the respective peak maxima in **Figure 2B**.

### pH Controlled Levan Production in Bioreactors

**FIGURE 3:**
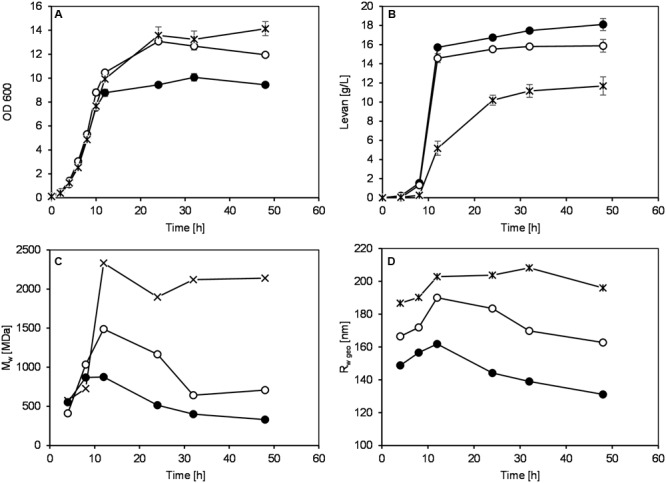
**Monitoring of OD600 (A)**, levan concentrations **(B)**, weight average molecular weight, M_w_ **(C)** and weight average geometric radius, R_wgeo_ **(D)** during levan production by *G. albidus* at different pH conditions: (●) pH 4.5, (∘) pH 5.5, and (×) pH 6.5. Data are average values ± SD, *n* = 3 **(A,B)** or are representative of at least two measurements from the same batch **(C,D)**.

In order to further understand how the change of pH affected the levan formation, levan productions by *G. albidus* were performed in bioreactors with controlled pH conditions at 4.5, 5.5, and 6.5, respectively. **Figure 3** shows the monitoring of absorbance (OD_600_, **Figure 3A**) and levan production (**Figure 3B**) at different pH conditions over 48 h. During the fermentation process, levan was isolated from the fermentation broth and analyzed by AF4-MALS-UV to determine the weight average molar mass (**Figure 3C**) and weight average geometric radius (**Figure 3D**) of these samples.

The increases of OD of the fermentation broth from three pH conditions were quite similar in the first 10 h, indicating a similar growth of *G. albidus* (**Figure 3A**). At 12 h, differences in the OD were observable and later became more apparent. Since there were big differences in the amounts of levan produced under different pH conditions (**Figure 3B**), the OD values after 12 h could be largely influenced not only by the cell density but also by the quantity and possibly the size of the produced levan particles. Levan production was the lowest at pH 6.5, having a final concentration at 48 h of 11.68 ± 0.95 g/L compared to 18.11 ± 0.63 and 15.88 ± 0.66 g/L at pH 4.5 and 5.5, respectively. According to the results, pH 4.5 revealed to be the best pH condition to obtain the highest levan amount.

**FIGURE 4:**
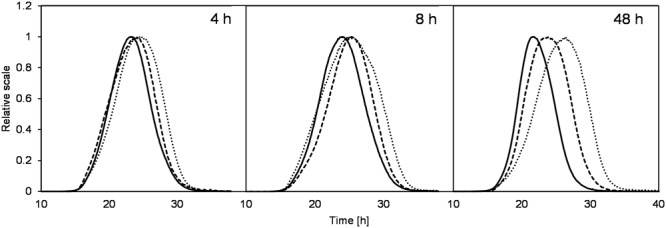
**Comparison of retention times from the LS signals (90°) of levans produced at pH 4.5 (solid line), 5.5 (dashed line), and 6.5 (dotted line) during the beginning (4 and 8 h) and end of fermentation (48 h)**.

Although the levan concentrations were higher if produced at constant pH 4.5, the weight average molar mass (M_w_) and size (R_wgeo_) of these levan samples were the smallest (**Figures 3C,D**, respectively). On the other hand, levan produced at pH 6.5 was the biggest in size but lowest in the quantity. In addition to the different levan sizes produced at different pH, there was also slight changes of the mass and size of levan formed during the fermentation process within one constant pH condition. During the first 12 h, the size and mass of levan particles increased slightly but later reduced to a different degree, especially at pH 4.5, while levan produced at pH 6.5 was relatively stable in its molecular size and mass. **Figure 4** displays the LS signals of levans produced under three pH conditions at 4, 8, and 48 h, in which the elution time of the separated fractions and the peak maxima of the levan size distributions were the highest at pH 6.5 and were the lowest at pH 4.5. At 48 h, the distance between the retention time of levan from each pH condition was also greater, confirming the more distinct variations in the levan size during levan production at different pH conditions.

### Effects of Differently Produced Levans on the Volume and Crumb Hardness of Gluten-Free Breads

**FIGURE 5:**
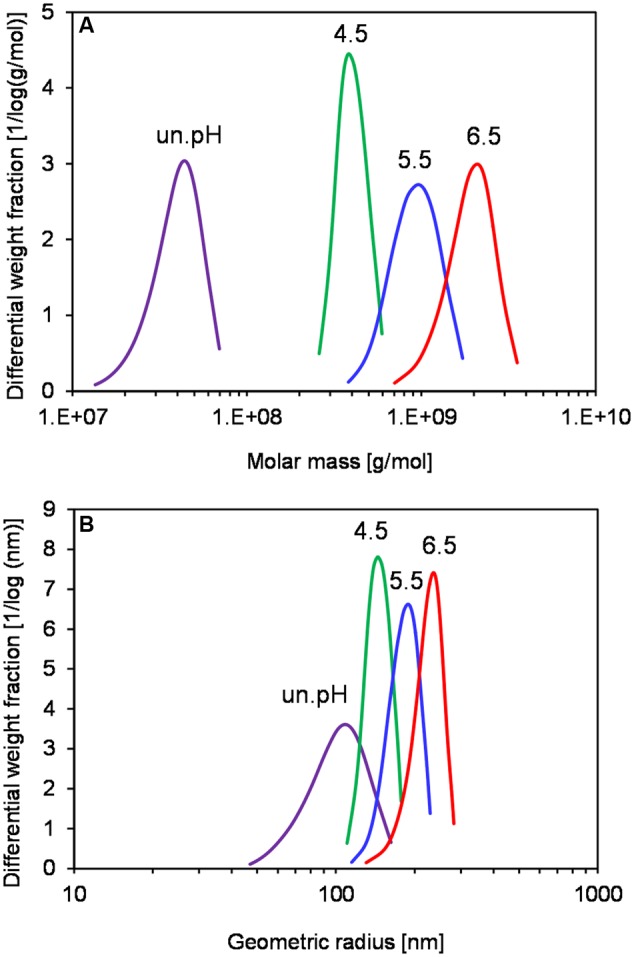
**Distribution of molar mass (A)** and geometric radius **(B)** of isolated levan from different production pH at 32 h. Data are representatives of at least two measurements.

In order to investigate if levans produced at different pH conditions exhibit different functional properties, an exploratory experiment on the levan application in GF baking was performed. Due to the lack of structural-building gluten proteins, GF breads generally have poor quality (low water binding capacity of doughs) and can solely be produced by addition of hydrocolloids such as polysaccharides. Levans were recovered at 32 h by *G. albidus* without pH control in shake flasks (un.pH) or at pH 4.5, 5.5, and 6.5 in bioreactors, and were incorporated into the plain buckwheat bread recipe. **Table 1** compares the cell count, levan concentrations and characteristics of each isolated levan, which were subsequently used for baking. Similar to the results shown in Section “pH Controlled Levan Production in Bioreactors,” the highest levan concentration at 32 h was achieved when controlling the pH at 4.5, followed by pH 5.5 and 6.5, respectively, while the highest molar mass and size of levan were obtained in reverse order. Levan produced in shake flasks without pH control, in which the pH of fermentation broth reduced from initially 6.2 to ca. 3 at 32 h, on the other hand, had the lowest concentration and the smallest mass and size. Due to the similar size distributions, the levan from this uncontrolled pH condition could represent the other isolated levans used for baking in other studies (Jakob et al., 2012b; Rühmkorf et al., 2012b). The differences in the size and mass of these samples were also compared in **Figure 5**, where the distributions of molar mass (**Figure 5A**) and geometric radius (**Figure 5B**) of the four isolated levans used in baking are depicted. While the R_wgeo_ of the four levan samples were overlapping to a certain extent, the M_w_ of levan from uncontrolled pH were far smaller than those produced at pH 4.5–6.5.

**Table 1:** Production of isolated levan from different pH conditions at 32 h used in baking.

Levan at pH	Cell count × 10^9^ [CFU/mL]	Levan [g/L]	Levan analysis				
			Mw [MDa]	Mn [MDa]	Polydispersity [–]	Rw geo [nm]	Rn geo [nm]
un.pH	0.12 ± 0.02	8.98 @ 1.18	41.0	37.0	1.108	103.6	95.3
4.5	2.07 ± 0.06	16.88 @ 1.19	405.8	391.8	1.036	144.8	142.0
5.5	2.83 ± 0.68	14.85 @ 1.25	959.0	870.1	1.102	180.9	173.3
6.5	3.64 ± 0.75	9.70 @ 2.98	1986.2	1796.2	1.106	223.2	213.0

*Cell count and levan data are average of at least two batches. Levan analysis data are representative from at least two measurements.*

*un.pH = uncontrolled pH.*

**FIGURE 6:**
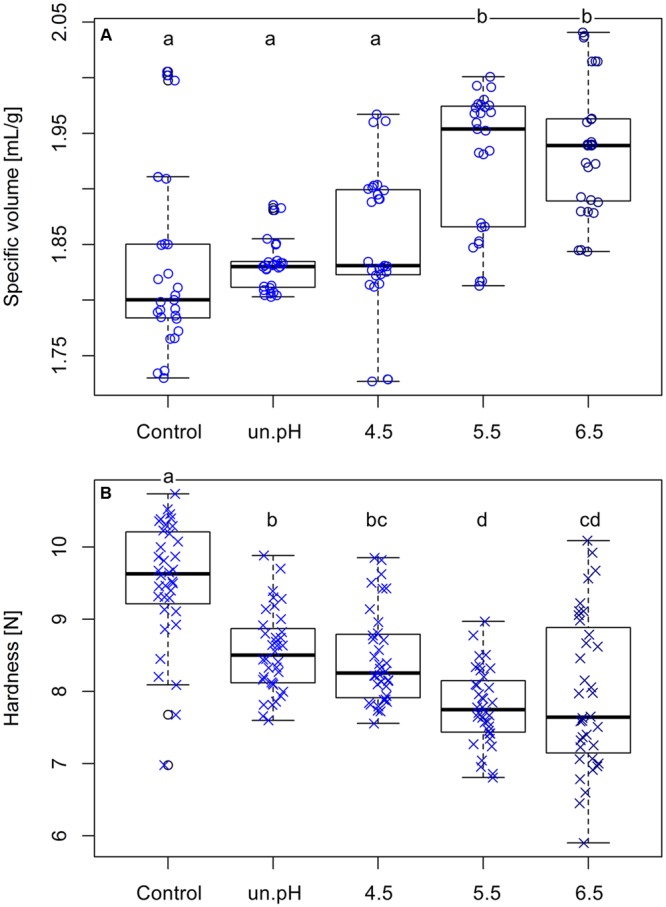
**Comparisons of the loaf specific volume (A)** and crumb hardness **(B)** of control breads and breads with 1% addition of isolated levan produced at different pH conditions. Data are from three separated bakings. Different letters indicate significant differences among treatments (*p* < 0.05).

After a confirmation that these levans were different in their mass and size, they were used in the baking of plain buckwheat breads to observe the effects on bread characteristics. Breads with 1% (flour base) addition of these isolated levans were analyzed and their specific loaf volumes and crumb hardness were compared to the control breads. The specific volumes of breads increased from 1.829 ± 0.079 mL/g in breads without levan (control) up to 1.937 ± 0.060 mL/g when 1% isolated levan from pH 6.5 was used. Although there was no significant difference in the volumes of control and breads with levan from uncontrolled pH and pH 4.5, an increasing trend was noticed, while a significant increase (*p* < 0.001) was observed with levan from pH 5.5 and 6.5 (**Figure 6A**). A more noticeable effect of levan with different sizes was detected on the hardness of bread crumbs (**Figure 6B**), in which buckwheat breads with the addition of larger size levan (pH 5.5 and 6.5) had the softest bread crumbs (7.964 ± 2.062 N: pH 6.5) compared to the levan with the smallest size from uncontrolled pH (8.546 ± 0.567 N) or control breads (9.522 ± 0.841 N). Since the same amounts of isolated levans were added in the recipe, the differences in the bread characteristics (volume and crumb hardness) could be entirely related to the variations in the molar mass and size of these levan samples. Nevertheless, breads with isolated levan produced at pH 5.5 and 6.5 were statistically similar, indicating a possible upper limit effect of levan size on the buckwheat bread quality.

## Discussion

Due to the potentials of bacterial EPS in several applications, the productions of EPS by different strains of bacteria and other microorganisms have been widely studied. Generally, the fermentation process and/or medium formulations were optimized to obtain the highest EPS concentration or yield (Chi and Zhao, 2003; Rühmkorf et al., 2012a; Qiang et al., 2013; Zhao et al., 2013; Ma et al., 2014) while some even utilized the metabolic engineering strategies to improve the EPS production (Senthilkumar et al., 2004; Feng et al., 2015). Nonetheless, few studies have focused on the possible changes of EPS size produced by one specific strain especially during the production process (Euzenat et al., 1997; Abdel-Fattah et al., 2005; Wu et al., 2013; Raga-Carbajal et al., 2016), which may finally influence the EPS functionality for various applications. In this study, the change of levan size produced by *G. albidus* was investigated, in which its size and subsequently its properties could be controlled by pH, since the environmental pH usually fluctuates to a great extent in the acid forming bacteria.

### Changes of Levan Size and Mass during Production

The monitoring of levan size and mass during the levan production in shake flasks (uncontrolled pH) by *G. albidus* confirmed earlier findings on the reduction of M_w_ and R_geo_ of *in situ* produced levan (Ua-Arak et al., 2016, 2017). The levan size decreased even though the levan concentration increased, suggesting that more levan of smaller size was produced whilst the already-synthesized levans were also hydrolyzed, resulting in the decline of average molar mass and radius along the fermentation process. During the fermentation by *G. albidus* TMW 2.1191, which expresses the levansucrase constitutively (Jakob, 2014), there were constant variations in the liquid culture such as substrate (sucrose), organic acids (acetic and gluconic acids) and pH. Since pH is one important factor affecting the structure and activity of enzymes, such a constant change of pH during AAB fermentation could indisputably influence the function of levansucrase. This change of levan size produced at different pH was later confirmed when the levan production was performed in a controlled pH condition (see pH Controlled Levan Production in Bioreactors), demonstrating that the reduction of pH during the levan production by AAB was the key factor regulating the size of levan being produced during the bacterial fermentation.

In addition to the formation of smaller levan at lower pH by levansucrase, the shift of levan size distribution toward the smaller range at longer fermentation time (**Figure 2B**) revealed that the existing larger levans produced at earlier time points were also hydrolyzed. The hydrolysis of the already-synthesized levan could result from a combination of factors such as enzymatic activities (Tanaka et al., 1978; Vigants et al., 2013) and acid hydrolysis or pH instability (Runyon et al., 2014; Sarilmiser et al., 2015). As reported for levansucrases from other bacterial species such as *Z. mobilis* (Yanase et al., 1992) and *B. subtilis* (Tanaka et al., 1978), levansucrases exhibit not only the fructosyltransferase activity, which transfers a fructose molecule from sucrose to the growing chain of levan (levan synthesis), but also an intrinsic levanase activity that releases a fructosyl moiety of a levan chain if water is used as acceptor (levan hydrolysis). The relation between transferase and hydrolysis activities of levansucrases can be influenced by parameters such as sucrose concentration, temperature, pH (Yanase et al., 1992; Vigants et al., 2013; Santos-Moriano et al., 2015) and possibly the branching point on the levan substrate (Méndez-Lorenzo et al., 2015), all of which could eventually contribute to the final molecular size distribution of levan in the liquid culture. An example of levan degradation by hydrolysis activity of levansucrase has been shown in the work of Ozimek et al. (2006), which occurred when the sucrose substrate depleted at high levansucrase activity isolated form *Lactobacillus (L.) reuteri* 121 (Ozimek et al., 2006).

Besides the levan hydrolysis by levansucrase, the enzyme levanase might also be responsible for the levan hydrolysis during the bacterial fermentation. For example, the expression of levanase gene from *Ga. diazotrophicus* was induced when glucose was depleted or when fructose concentration was lower than 0.44 mM (Menéndez et al., 2009). Although not fully characterized, a putative levanase (glycoside hydrolase family 32) encoding gene is strictly conserved in the genus *Gluconobacter* including *G. albidus* (data from BLASTP search at NCBI), which might be necessary for cell survival in case of carbon starvation (Öner et al., 2016). Nevertheless, since the total amount of levan was still relatively stable during 48 h of levan production as shown in Section “Levan Production in Shake Flasks without pH Control,” the levanase activity might not yet be fully active or there was a similar rate of levan synthesis and hydrolysis at this stage of fermentation.

Due to the formation of organic acids during the AAB fermentation as well as the pH reduction to a minimum of pH 2.8, it was possible that the reduction of levan size and mass was partially due to spontaneous acid hydrolysis. At 40°C, the hydrolytic degradation of levan has been reported in the commercial levan from *Z. mobilis* at pH lower than 5.5 (Runyon et al., 2014), while no acid hydrolysis was observed at 30°C even at pH 3.7 (Bekers et al., 2005). Since the pH in Section “Levan Production in Shake Flasks without pH Control” was reduced to around pH 2–3, the acid hydrolysis of levan at the mild temperature of 30°C might still occur.

Varying pH conditions influenced both the levan production and its molecular size. The difference in the levan production could be from the differences in the levansucrase activity as well as enzyme concentration at varying pH conditions. pH has always been one of the key factors affecting the levansucrase activity, in which the optimal pH of the enzyme differs depending on the source of levansucrase (Öner et al., 2016). For example, the highest levansucrase activity was found at pH 5.0 in levansucrase from *Z. mobilis* (Jang et al., 2001), *Ga. diazotrophicus* (Hernandez et al., 1995) and *G. albidus* (Jakob and Vogel, 2015), at pH 6.0 from *Bacillus* sp. TH4-2 (Ben Ammar et al., 2002) and at pH 8.0 from *B. subtilis* NRC1aza (Esawy et al., 2013), respectively. In addition to the influence of pH on enzyme activity, the amount of enzyme being synthesized and/or secreted in the liquid medium might also be different. Abdel-Fattah et al. (2005) reported the influence of enzyme concentration on the levan production by levansucrase from *B. subtilis* NRC33a, in which increasing levansucrase concentration synthesized higher amounts of levan with relatively similar molecular weight (Abdel-Fattah et al., 2005). Accordingly, assuming that the production and constitutive secretion of levansucrase by *G. albidus* in liquid culture was higher at pH 4.5 than at 6.5, there could be higher concentrations of levansucrases at certain pH, resulting in higher amount of produced levan and a possible concomitant faster consumption of the substrate sucrose, which again would increase the possibility to partially hydrolyze levan chains due to the intrinsic levanase activity of levansucrases as mentioned above.

### Influence of Differently Produced Levans on the Quality of GF Breads

Levan has been of interest for the improvement of GF bread quality since it is produced naturally by some indigenous sourdough bacteria such as *L. sanfranciscensis* (Korakli et al., 2001) and *L. reuteri* (Schwab et al., 2008). The positive effect of levan on wheat bread quality was shown before by Jakob et al. (2012b), where an increase of loaf volume and a reduction of bread staling rate were observed by the addition of isolated levans from different strains of AAB. Additionally, Rühmkorf et al. (2012b) demonstrated the contribution of branching position and size of EPS to the structural improvement of GF bread. Nevertheless, EPS from these earlier studies were produced from different bacterial strains and were sometimes a mixture of different types of EPS, which could to some extent influence the bread characteristics. In this study, levan was produced by *G. albidus* at varied pH conditions to obtain the same levan with only differences in size and mass, in which the stronger positive effect was observed when using larger isolated levan. The size and mass distributions of levan (**Figure 5**) shows that the differences in radii of each levan samples were smaller than the differences in molar mass, indicating a more compact nature of levan molecules at larger size, as observed before in levan from other AAB (Jakob et al., 2013), *Bacillus* sp. (Arvidson et al., 2006) and *Streptococcus salivarius* (Newbrun et al., 1971).

Although the mechanism of levan on the improvement of bread quality is still unclear, the micro-gel characteristic of the spherical levan molecules might bind water individually (Kitamura et al., 1994) and/or interact with water and flour particles (Rühmkorf et al., 2012b), eventually reducing the crumb hardness and increase the loaf volume. The influences of levan from three different bacterial strains with varied molecular weights but similar branching on the rheological properties of levan aqueous solutions were demonstrated before by Benigar et al. (2014), whereby a pseudoplastic behavior (shear thinning) of levan solution with higher molecular weight was observed at a lower concentration than the smaller levan, and the aqueous solutions of larger levan were more viscous than the smaller ones at the same concentration (Benigar et al., 2014). In this study, larger levans affected more positively the bread characteristics than smaller levans. This might be due to the stronger influences of high M_w_ levan on the viscoelastic properties of the bread batter, and subsequently on the better quality of GF bread. The sourdough production is generally a natural fermentation process with the end pH ranges from 3.5 to 4.0 (Arendt et al., 2007), subsequently, the *in situ* produced levan in the sourdough would probably have decreasing size in accordance to the pH reduction. The smaller levan due to low pH in sourdough might be one of the reasons that little or no significant effect on the bread characteristics was observed in some studies (Kaditzky et al., 2008; Schwab et al., 2008), signifying the influence of production pH on the function of levan in the end application.

This utilization of levan for the improvement of GF bread quality was an example to illustrate the impact of levan size and mass on its function in a baking application. Generally, a typical optimization of levan production would focus on the amount of levan being produced, i.e., at pH 4.5 by *G. albidus* in this work, while the changes of the levan size at varying optimizing parameters were overlooked. This may lead to a loss in the opportunity of utilizing levan in a potential application, which may be possible only with the levan of a particular size, as demonstrated by the significant improvement of breads with levan produced from pH 5.5 and 6.5. Several environmental conditions have been described to be responsible for the variations in the levan production and the molecular size and mass of the produced levan. Levan concentration is influenced mainly by sucrose concentration, and also by other factors such as incubation time, temperature, initial pH, presence of yeast extract and Mg^2+^, NaCl, thiamine content, etc. (Ernandes and Garcia-Cruz, 2011; Santos et al., 2014; Silbir et al., 2014; Zhang et al., 2014; Santos-Moriano et al., 2015; Sarilmiser et al., 2015).

Since the molar mass and size of EPS influence their characteristics and eventually determine their functions, understanding the change of levan size and mass under a certain culture condition will give more insights into the production of levan. As a result, the levan production with the desired characteristics is possible by tailoring the molecular mass of choice. This study demonstrates the changes of levan molecular size and mass influenced by the pH condition, and how a simple control of pH during the levan production could be used to produce the levan with specific size ranges for a specific application such as in GF baking.

## Author Contributions

TU-A: performed and planned the main experimental work presented in this manuscript and wrote the main text of the manuscript. FJ: was involved in some experimental work, planning the experimental setup and in writing the manuscript. RV: was involved in planning the experimental setup and writing the manuscript.

## Conflict of Interest Statement

The authors declare that the research was conducted in the absence of any commercial or financial relationships that could be construed as a potential conflict of interest.
